# Food insecurity associated with self-reported mental health outcomes in Peruvian households during the COVID-19 pandemic

**DOI:** 10.3389/fnut.2022.1005170

**Published:** 2022-11-04

**Authors:** Maria M. Anampa-Canales, Salomón Huancahuire-Vega, Edda E. Newball-Noriega, Wilter C. Morales-García, Cesar Augusto Galvez

**Affiliations:** ^1^Department of Public Health, Postgraduate School, Universidad Peruana Unión (UPeU), Lima, Peru; ^2^Department of Basic Sciences, Faculty of Health Sciences, School of Human Medicine, Universidad Peruana Unión (UPeU), Lima, Peru

**Keywords:** food insecurity, perceived mental health, COVID-19, pandemic, self-report, Peru

## Abstract

**Background:**

The global pandemic of COVID-19 and the social distancing efforts implemented worldwide to limit its spread have disrupted the economy, increased food insecurity, and mental health problems.

**Objectives:**

The aim of this study was to determine the association between food insecurity and mental health outcomes (stress, depression, and anxiety) in Peruvian households during the COVID-19 pandemic.

**Materials and methods:**

A cross-sectional investigation was conducted with 525 participants of both sexes (68% women), over 18 years of age and from the three geographic regions of Peru: Coast (54.9%), Highlands (28.4%), and Jungle (16.8%). The data were collected during the year 2021, between July 6 and September 22 through a self-administered online survey designed to assess socio-demographic, socioeconomic, food insecurity, and mental health outcomes (depression, anxiety, and stress).

**Results:**

The majority of households (71.4%) experienced some degree of food insecurity. Mild food insecurity was the most frequent, affecting 49.1%, followed by moderate 15.4%, and severe 6.9%. Regarding mental health outcomes, 24.8% manifested depression, 26.7% anxiety, and 15.3% stress. With respect to the association between the level of food insecurity and anxiety, this was highly significant (*p* < 0.01). Households with mild, moderate and severe food insecurity are 2.04, 4.5, and 10.44 times, respectively, more likely to have moderate-severe anxiety. On the other hand, the mild food insecurity was not associated with moderate-severe depression. In contrast, households with moderate and severe food insecurity are 2.8 and 5.7 times, respectively, more likely to have moderate-severe depression. Finally, households with moderate food insecurity are 5.9 times more likely to have moderate-severe stress, and households with severe food insecurity are 8.5 times more likely to have moderate-severe stress, both having a highly significant association (*p* < 0.01).

**Conclusion:**

In conclusion, during the second wave of the COVID-19 pandemic in Peru, household food insecurity was independently associated with worse mental health outcomes. Monitoring of both food insecurity and mental health will be important as the COVID-19 pandemic continues.

## Introduction

The inalienable right of everyone to have access to safe and nutritious food was reaffirmed in 1996 during the World Food Summit. At this event, a global commitment was made to eliminate hunger and malnutrition and to ensure sustainable food security for the entire population (1).

Food security is defined as the fundamental right that is fulfilled when people have physical, social, and economic access at all times to sufficient, safe and nutritious food to meet their needs and preferences for an active and healthy life (2). In contrast, food insecurity is defined as limited access to adequate food due to social, economic, or other constraints (3). This is an urgent public health and social problem, which varies in degree and impact on individuals and social groups.

Since January 2020, the World Health Organization (WHO) has declared a global pandemic of coronavirus disease, known as COVID-19. According to the Food and Agriculture Organization (FAO), since the beginning of this pandemic, one out of three people in the world has lacked access to adequate food, which represents 2.37 billion people (2). Several studies show that moderate and severe food insecurity worldwide has increased due to the COVID-19 pandemic, for example, an increase of 17 million food insecure Americans has been projected in 2020 (4). The same occurred in the Latin American population, where moderate or severe food insecurity increased from 30.1 to 39.2% (5).

Although food security is not routinely measured in national health surveys in Peru, some reports prior to the pandemic indicated moderate/severe prevalence rates between 37.5% of food insecurity without hunger to 77.2% of moderately food insecure (6–8). These prevalence rates of food insecurity in the country was worsened by the arrival of the pandemic. In a study of households in low-income urban areas of Peru, 46.9% of households experienced moderate household food insecurity and 4.1% experienced severe food insecurity (9). Unemployment and loss of income due to COVID-19 would be the probable causes of the increase in food insecurity in Peruvian households (10). According to a survey conducted by the National Institute of Statistics and Informatics to households in Metropolitan Lima and Callao in May 2020, 14% of households stated that they were unable to buy food with protein content such as meat, fish, and eggs, the main cause being lack of financial means (11). Similar facts have been occurring in the region, where the pandemic has increased hunger, poverty, and unemployment (12).

On the other hand, the pandemic has increased the alterations of the emotional state, these alterations of the mental state negatively affect quality of life (13, 14), as well as physical and mental health (15). The COVID-19 pandemic has triggered many people to be affected emotionally, experiencing depression, anguish, and stress, putting their health and general wellbeing at risk (16). In Peru, 28.6% of the population presented depressive symptoms and the age group with the greatest depressive affectation was 18–24 years old (17). Likewise, 59.7% of Peruvians suffered from stress due to the pandemic (18).

Studies in several countries during the early period of the COVID-19 pandemic have shown an independent association between household food insecurity and altered mental health states (19, 20). In addition, a meta-analysis suggests that food insecurity has a significant effect on the likelihood of being stressed or depressed (21). In this sense, these associations have not yet been verified in the population with more deaths per million inhabitants worldwide due to COVID-19, which led to estimate that life expectancy in Peru will decrease by more than 2 years in 2020 (22). This, in addition to the poor management of the pandemic by its authorities (23). Therefore, the objective of the study is to determine the association between food insecurity and emotional state alterations (stress, depression, and anxiety) in Peruvian households during the COVID-19 pandemic.

## Materials and methods

### Population and study design

Cross-sectional design study through a structured survey of adult men and women aged ≥18 years, belonging to Peruvian households. Data were collected in the year 2021, between July 6 and September 22, during the final part of the second wave of the COVID-19 pandemic. During this period, the country was still in a health emergency, although many of the restrictive measures had been suspended. The study was done with participants from the three regions of the country, coast, Andes, and jungle. Sampling was for convenience. For the coast region, the questionnaire was distributed through the social networks of the University to which the authors belong. In addition, the authors distributed it through their social networks. For the Andes and jungle regions, two people living in each region were hired to disseminate the study through email, Facebook, Instagram, and WhatsApp. All participants were invited to complete a structured online questionnaire using the Microsoft Forms web survey platform. To ensure the criteria for inclusion in the research, these criteria were included in the informed consent, as options in which the participant agreed or did not agree to continue with the completion of the form. A total of 540 responses were received. After excluding those under 18 years of age, incomplete surveys, and respondents from other countries, the final data set included 525 participants.

### Ethical aspects

This study followed the international ethical standards found in the Declaration of Helsinki (2000). All procedures involving human subjects were approved by the Ethics and Research Committee of the Universidad Peruana Unión (No 2021-CE-EPG-000014). A brief description of the study and its objective, as well as informed consent, were provided on the first electronic page containing the invitation to participate in the survey. All subjects gave their consent to participate in the study after clicking on the “accept” icon, meaning that they accepted the terms of the informed consent.

### Measuring instruments

Data collection was carried out through a single self-administered structured digital questionnaire developed by the authors using Microsoft Forms. The questionnaire contained questions on sociodemographic aspects (age, sex, marital status, employment status, region, type of family, number of children, and socioeconomic status). To assess socioeconomic status, the socioeconomic status scale was used, which consists of five levels: high, medium, upper low, lower low, and marginal (A, B, C, D, and E, respectively). This scale has five dimensions: (1) Education of the head of the family and spouse, (2) Characteristics of the dwelling, (3) Access to health, (4) Economic income of the family, and (5) Overcrowding. The categorization of socioeconomic status was obtained based on the following scores: A (33 or more), B (27–35), C (21–26), D (13–20), and E (5–12) (24). The Latin American and Caribbean food security scale (ELCSA) was used to assess food insecurity, and the depression, anxiety, and stress abbreviated scale (DASS-21) was used to assess mental health outcomes (depression, anxiety, and stress).

#### Latin American and Caribbean food security scale

This instrument was developed by the ELCSA Scientific Committee and is used by the FAO of the United Nations. The ELCSA Scientific Committee developed a formal process of interactive and iterative consultation, between 2007 and 2011, to promote the development of a single scale for the measurement of food insecurity in households in Latin America and the Caribbean. This process included two regional conferences on household food insecurity measurement, the application of consensus versions of the ELCSA in different countries, and various regional workshops on harmonization and statistical analysis. The ELCSA was developed from the US Household Food Security Supplement Module (HFSSM), the Brazilian Food Insecurity Scale (EBIA), the Lorenzana Scale validated and applied in Colombia, and also taking into account the Food Insecurity and Access Scale developed by the United States Agency for International Development (25). The instrument has good psychometric properties that demonstrate reliability and validity (25–27). Typical Cronbach’s alpha values in validation studies of the ELCSA (0.91–0.96) suggest that this instrument has a high degree of internal consistency in different socioeconomic and cultural contexts (27). The scale consists of 3 dimensions: (1) Quantity and quality of food; (2) Uncertainty/Anxiety about food access or availability, and (3) Socially unacceptable means of food acquisition and distribution. It is divided into two sections: the first section with eight questions (questions 1–8) referring to various situations that lead to food insecurity, experienced in households and adults in those households; and in the second section with seven questions (questions 9–15), referring to conditions affecting children under 18 years of age in the household. Therefore, in households where there are children under 18, respondents answer all 15 items of the ELCSA, and in those households where there are only adults, only the first eight items are applied. The ELCSA allows us to classify food insecurity into three levels: (a) Mild food insecurity, (b) Moderate food insecurity, and (c) Severe food insecurity (28). To calculate the score for the classification of the level of food insecurity, one point will be assigned for each “Yes” answer and zero for each “No” answer. At the end, the affirmative answers to the ELCSA questions are added up, and the classification of the levels of food insecurity will be made using the scores: 0 (food security), 1–5 (mild insecurity), 6–10 (moderate insecurity), 11–15 (severe insecurity) for households composed of adults and children under 18 years of age. For households composed only of adults the cut-off points are: food security (0), mild insecurity (1–3), moderate insecurity (4–6), severe insecurity (7–8). In the results we are denominating as “Normal” level, those households that presented food security. The cut-off points were obtained from the Manual for the use and application of ELCSA (25).

#### Depression, anxiety, and stress abbreviated scale

The DASS-21 scale has 21 items, which is composed of three dimensions measuring depression, anxiety, and stress. The depression dimension (items 3, 5, 10, 13, 16, 17, and 21) assesses unpleasant emotions, hopelessness, sadness, loss of interest, and low self-esteem. The anxiety dimension (items 2, 4, 7, 9, 15, 19, and 20) assesses psychophysiological activation, autonomic arousal, and subjective experiences of anxiety. Finally, the stress dimension (items 1, 6, 8, 11, 12, 14, and 18) assesses the difficulty to be relaxed, nervous excitement, agitation, irritability, and impatience (29). All items have a Likert-type response format with four alternatives, which are ordered on a scale of 0 to 3 points. The response options available for answering this scale were: 0 (it has not happened to me), 1 (it has happened to me a little, or for part of the time), 2 (it has happened to me a lot, or for a good part of the time), and 3 (it has happened to me a lot, or most of the time) (30). The scale score is calculated with the sum of the scores of the items belonging to that scale and varies between 0 and 21 points. According to the score of each domain, the severity of the disturbance is established. The higher the score, the greater the severity of depression, anxiety, and stress (29). The instrument used was validated in a Peruvian sample whose reliability was Cronbach’s alpha 0.975 (31). With our database, Cronbach’s alpha for the depression, anxiety, and stress subscales were: 0.923, 0.895, and 0.933, respectively, which confirms the reliability of the measurement instrument. In the results, we divided the outcomes, which are anxiety, depression, and stress, into two levels: normal–mild and moderate–severe. In the normal–mild level, we considered the categories Normal and Mild of the DASS-21 Scale and for the Moderate-Severe level, we considered the categories Moderate, Severe, and Extremely Severe of the same scale.

### Data analysis

Data analysis was performed through the programming language R version 4.0.2. Depending on their categorical or numerical nature, the variables were described as absolute and relative frequencies (%) or mean and standard deviation (SD), respectively. For the comparative analysis between the categorized groups, the chi-square test was used. Then, to establish the independent relationship between food insecurity and anxiety, stress, or depression in the study population, Poisson regression models with robust variance were used. These regression models provided the PRc (crude prevalence ratio) and PRa (adjusted prevalence ratio) for each factor, with their respective 95% confidence intervals (95% CI). Adjustment was made for sex, age group, and socioeconomic status. A *p* < 0.05 was considered statistically significant in all analyses.

## Results

### General characteristics of population

Table 1 shows sociodemographic characteristics. A total of 525 adult participants over 18 years of age from Peruvian households were analyzed, with a greater distribution between 36 and 64 years of age (55.6%), and significant differences were found between men and women. Regarding marital status, 57.0% were married-cohabitant. Regarding employment status, 58.1% of the population reported not working or having a casual job. For the study, we considered collecting the sample from the three regions of Peru, with the coast being the most predominant with 54.9%. The 57.3% came from nuclear families and 41.7% belonged to socioeconomic level D.

**TABLE 1:** General characteristics of the population.

Variables	Total (*n* = 525)	Men (*n* = 167)	Women (*n* = 358)	*P*-value
Age				0.019*
18–35 years old	227 (43.2%)	58 (34.7%)	169 (47.2%)	
36–64 years old	292 (55.6%)	107 (64.1%)	185 (51.7%)	
>65 years old	6 (1.1%)	2 (1.2%)	4 (1.1%)	
Marital status				0.161
Married	189 (36.0%)	57 (34.1%)	132 (36.9%)	
Cohabitant	110 (21.0%)	29 (17.4%)	81 (22.6%)	
Divorced	19 (3.6%)	8 (4.8%)	11 (3.1%)	
Single	202 (38.5%)	73 (43.7%)	129 (36.0%)	
Widower	5 (1.0%)	0 (0.0%)	5 (1.4%)	
Employment status				<0.001**
Does not work	104 (19.8%)	10 (5.99%)	94 (26.3%)	
Stable job	220 (41.9%)	84 (50.3%)	136 (38.0%)	
Temporary employment	201 (38.3%)	73 (43.7%)	128 (35.8%)	
Region				0.049*
Coast	288 (54.9%)	81 (48.5%)	207 (57.8%)	
Highlands	149 (28.4%)	59 (35.3%)	90 (25.1%)	
Jungle	88 (16.8%)	27 (16.2%)	61 (17.0%)	
Family type				<0.001**
Extensive	90 (17.1%)	25 (15.0%)	65 (18.2%)	
Single parent	67 (12.8%)	16 (9.6%)	51 (14.2%)	
Nuclear	301 (57.3%)	93 (55.7%)	208 (58.1%)	
Single person	55 (10.5%)	33 (19.8%)	22 (6.2%)	
Reconstituted	12 (2.3%)	0 (0.0%)	12 (3.4%)	
Children under 5 years old				0.032*
None	324 (61.7%)	111 (66.5%)	213 (59.5%)	
1 child	122 (23.2%)	28 (16.8%)	94 (26.3%)	
2 children	30 (5.7%)	11 (6.6%)	19 (5.31%)	
3 children	14 (2.7%)	5 (3.0%)	9 (2.51%)	
4 children	3 (0.6%)	3 (1.8%)	0 (0.0%)	
>5 children	32 (6.1%)	9 (5.4%)	23 (6.4%)	
Socioeconomic status				0.217
A	16 (3.0%)	9 (5.4%)	7 (2.0%)	
B	88 (16.8%)	29 (17.4%)	59 (16.5%)	
C	198 (37.7%)	62 (37.1%)	136 (38.0%)	
D	219 (41.7%)	65 (38.9%)	154 (43.0%)	
E	4 (0.8%)	2 (1.2%)	2 (0.6%)	

Data presented as absolute and relative frequency (%).

*p < 0.05, **p < 0.01, statistically significant by chi-square.

### Prevalence of food insecurity and alterations in emotional state

**FIGURE 1:**
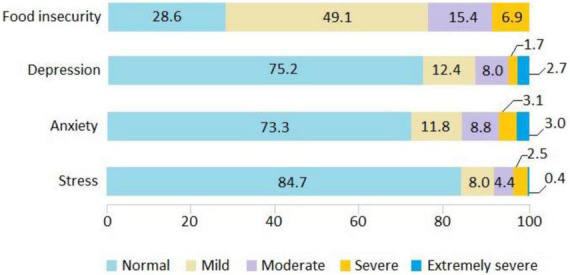
Prevalence of food insecurity and mental health outcomes in Peruvian households during the COVID-19 pandemic.

According to the results obtained from the 15 questions of the ELCSA, 71.4% of households experienced some degree of food insecurity. Mild food insecurity was the most frequent, affecting 49.1%, followed by moderate 15.4%, and severe 6.9%. Regarding mental health outcomes, 24.8% manifested depression, 26.7% anxiety, and 15.3% stress (Figure 1).

**FIGURE 2:**
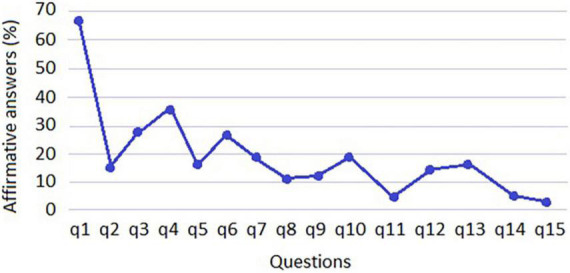
Percentage of affirmative responses by Latin American and Caribbean food security scale (ELCSA) questions.

The study’s finding also revealed that due to lack of money or other resources 65.7% of households were worried that food would run out, 15.0% ran out of food at some time in their households, 27.0% stopped having a healthy diet, 35.6% had a diet based on little variety of food, about 14.9% stopped eating breakfast, lunch or dinner, 28.0% ate less than they should eat, 19.6% felt hungry but did not eat, and 10.7% ate only once a day or stopped eating for a whole day. With respect to households with the presence of minors under 18 years of age, the study revealed that 11.4% of these minors stopped having a healthy diet, 18.9% had a diet based on little variety of food, 4.8% stopped eating breakfast, lunch, or dinner, 13,0% of children ate less than they should, 15.8% of households had to reduce the amount of food served to a child, 5.5% of children felt hungry but did not eat, and 3.8% of children ate only once a day or stopped eating for a whole day (Figure 2).

### Food insecurity and anxiety

The results indicate that sex is significant for anxiety (*p* < 0.01). According to the adjusted analysis, men are 51.0% less likely to have moderate-severe anxiety than women. Age and socioeconomic status showed no association. With respect to the association between the level of food insecurity and anxiety, this is shown to be highly significant (*p* < 0.01). As the level of food insecurity increases, the risk of moderate-severe anxiety increases. The results reveal that households with mild food insecurity are 2.04 times more likely to have moderate-severe anxiety, households with moderate food insecurity are 4.5 times more likely to have moderate-severe anxiety and households with severe food insecurity are 10.44 times more likely to have moderate-severe anxiety (Table 2). All these categories have increased risk compared to food security group.

**TABLE 2:** Association between level of food insecurity and anxiety.

	Anxiety				
Variables	N-M level (*n* = 471)	M-S level (*n* = 54)	*P*-value	PRc (95% CI)	PRa (95% CI)
**Sex**					
Women	313 (66.5%)	45 (83.3%)	0.018	Reference	Reference
Men	158 (33.5%)	9 (16.7%)		0.51 (0.30–0.87)*	0.38 (0.2–0.7)**
**Age**					
≤35 years	210 (44.6%)	23 (42.6%)	0.598	Reference	Reference
>35 years	261 (55.4%)	31 (57.4%)		0.89 (0.59–1.3)	1.06 (0.65–1.73)
**Socioeconomic status**					
A–B	51 (10.8%)	6 (11.1%)	0.491	Reference	Reference
C–D	400 (84.9%)	44 (81.5%)		0.94 (0.42–2.11)	0.63 (0.31–1.27)
E	20 (4.25%)	4 (7.41%)		1.58 (0.49–5.11)	0.67 (0.22–2.0)
**Food insecurity**					
Normal	144 (30.6%)	6 (11.1%)	<0.001	Reference	Reference
Mild	237 (50.3%)	21 (38.9%)		2.2 (1.5–7.0)*	2.04 (0.85–4.89)*
Moderate	67 (14.2%)	14 (25.9%)		4.8 (2.1–10.9)**	4.5 (1.78–11.3)**
Severe	23 (4.9%)	13 (24.1%)		8.3 (3.6–37.8)**	10.44 (4.26–25.6)**

Data presented as absolute and relative frequency (%). N-M, normal-mild; M-S, moderate-severe; PRc, crude prevalence ratio; PRa, prevalence ratio adjusted for all variables presented; 95% CI, 95% confidence interval.

*p < 0.05, **p < 0.01, statistically significant by Poisson regression with robust variance.

### Food insecurity and depression

It is observed that sex is significant for depression (*p* < 0.01). According to the adjusted analysis, men are 57.0% less likely to have moderate-severe depression compared to women. Age and socioeconomic status showed no association. The level of mild food insecurity is not associated with the development of moderate-severe depression. Instead, households with moderate food insecurity are 2.8 times more likely to have moderate-severe depression, (*p* < 0.05), while households with severe food insecurity are 5.7 times more likely to have moderate-severe depression, having a highly significant association (*p* < 0.01) compared to food security group (Table 3).

**TABLE 3:** Association between level of food insecurity and depression.

	Depression				
Variables	N-M level (*n* = 460)	M-S level (*n* = 65)	*P*-value	PRc (95% CI)	PRa (95% CI)
**Sex**					
Women	304 (66.1%)	54 (83.1%)	0.009	Reference	Reference
Men	156 (33.9%)	11 (16.9%)		0.44 (0.23–0.81)**	0.43 (0.23–0.81)**
**Age**					
≤35 years	194 (85.5%)	33 (14.5%)	0.190	Reference	Reference
>35 years	266 (89.3%)	32 (10.7%)		0.74 (0.47–1.2)	0.73 (0.47–1.15)
**Socioeconomic status**					
A–B	52 (11.3%)	5 (7.69%)	0.571	Reference	Reference
C–D	388 (84.3%)	56 (86.2%)		1.43 (0.6–3.44)	1.07 (0.46–2.54)
E	20 (4.35%)	4 (6.15%)		1.9 (0.56–6.48)	1.09 (0.33–3.69)
**Food insecurity**					
Normal	141 (30.7%)	9 (13.8%)	0.001	Reference	Reference
Mild	227 (49.3%)	31 (47.7%)		1.88 (0.91–4.1)	1.9 (0.9–3.9)
Moderate	67 (14.6%)	14 (21.5%)		2.9 (1.3–6.4)**	2.8 (1.2–6.3)*
Severe	25 (5.43%)	11 (16.9%)		5.1 (2.3–11.4)**	5.7 (2.5–13.0)**

Data presented as absolute and relative frequency (%). N-M, normal-mild; M-S, moderate-severe; PRc, crude prevalence ratio; PRa, prevalence ratio adjusted for all variables presented; 95% CI, 95% confidence interval.

*p < 0.05, **p < 0.01, statistically significant by Poisson regression with robust variance.

### Food insecurity and stress

Gender is also significant for stress (*p* < 0.05). Men are 85.0% less likely to have moderate-severe stress compared to women. As for age, a significant association is shown here (*p* < 0.05), with those older than 35 years being 54.0% less likely to develop moderate-severe stress. On the other hand, the level of mild food insecurity is not associated with the development of moderate-severe stress. Instead, households with moderate food insecurity are 5.9 times more likely to have moderate-severe stress, and households with severe food insecurity are 8.5 times more likely to have moderate-severe stress, both having a highly significant association (*p* < 0.01) compared to food security group (Table 4).

**TABLE 4:** Association between the level of food insecurity and stress.

	Stress				
Variables	N-M level (*n* = 495)	Level M-S (*n* = 30)	*P*-value	PRc (95% CI)	PRa (95% CI)
**Sex**					
Women	330 (66.7%)	28 (93.3%)	0.004	Reference	Reference
Men	165 (33.3%)	2 (6.67%)		0.12 (0.03–0.45)**	0.15 (0.03–0.67)*
**Age**					
≤35 years	212 (42.8%)	21 (70.03%)	0.008	Reference	Reference
>35 years	283 (57.2%)	9 (29.07%)		0.44 (0.24–0.84)*	0.36 (0.17–0.76)*
**Socioeconomic status**					
A–B	55 (11.1%)	2 (6.67%)	0.254	Reference	Reference
C–D	419 (84.6%)	25 (83.3%)		1.6 (0.39–6.6)	1.09 (0.29–4.08)
E	21 (4.24%)	3 (10.0%)		3.56 (0.63–20.0)	1.75 (0.31–9.76)
**Food insecurity**					
Normal	147 (29.7%)	3 (10.0%)	0.002	Reference	Reference
Mild	246 (49.7%)	12 (40.0%)		2.5 (0.85–7.2)	2.07 (0.62–6.84)
Moderate	71 (14.3%)	10 (33.3%)		5.6 (1.8–16.7)**	5.9 (1.7–20.5)**
Severe	31 (6.26%)	5 (16.7%)		5.2 (1.5–18.4)*	8.5 (2.1–34.0)**

Data presented as absolute and relative frequency (%). N-M, normal-mild; M-S, moderate-severe; PRc, crude prevalence ratio; PRa, prevalence ratio adjusted for all variables presented; 95% CI, 95% confidence interval.

*p < 0.05, **p < 0.01, statistically significant by Poisson regression with robust variance.

## Discussion

The COVID-19 pandemic has had a major impact on household food security globally. The findings demonstrate that food insecurity impacted a large proportion in Peruvian population, and food insecurity was in turn associated with different forms of mental health distress. Despite more than 2 years having passed since the onset of the pandemic in the country, we continue to see high rates of both food insecurity and mental health problems.

Previous studies have shown the increase in food insecurity due to the COVID-19 pandemic, in addition, the measures implemented by governments to contain this health crisis, such as confinement and social isolation, have exacerbated food insecurity (32, 33). Although many of these measures have been suspended, high levels of food insecurity are still evident. This is what was found in this study, where 71.4% of Peruvian households presented some degree of food insecurity. Recent studies of populations in other countries of the region show similar prevalence rates (34). Poverty, concern about unemployment and loss of income due to COVID-19 are the probable causes of the increase in food insecurity in Peruvian households (9). A national study showed that the country’s positive economic growth from 2005 to 2015 was interrupted and even declined by 0.8% during the pandemic (10). Poverty, which has not been substantially reduced in recent years, increased dramatically with the current health crisis, reaching 30.1%, which means an increase of 9.9% between 2019 and 2020 (11). In addition to these economic indicators, the Peruvian economy is characterized by its high informality, which reaches 69.2% (35). In relation to employment, a study conducted in the southern region of the country showed that 39% of households would have lost at least one job during the pandemic (36). In addition, most of the participants in this study (80.2%) were in socio-economic levels C, D, and E. This information together would explain the high prevalence of food insecurity in Peruvian households.

Regarding the characteristics of food insecurity, the study showed that 65.7% of Peruvian households expressed concern about food shortages during the pandemic. This is reaffirmed by other studies that show very similar results (37). The study also reports that 27% of households did not have a healthy diet, and 35% had little variety of food. In addition to the omission of these in some meal time (breakfast, lunch, or dinner), which generates insecurity in terms of quantity and nutritional quality. In this regard, FAO in its analysis and responses in Latin America and the Caribbean to the effects of the COVID-19 pandemic on food systems, has pointed out a high intake of sugars, sodium, and saturated fats in the population, in contrast to access to a balanced diet, which puts people’s health at risk (38). In addition, the lack of nutritional quality and access to a healthy diet would weaken the immune system of the population and leave them susceptible to contracting or developing severe forms of the SARS-CoV-2 transmitted disease (39).

In relation to mental health, considerable prevalence rates of depression, anxiety, and stress were observed in the population. Interestingly, it was observed that the gender variable is very significant for the development of emotional alterations, with men being less likely to have depression, anxiety, and stress at the moderate-severe level compared to women. This is corroborated by other studies that concluded that female gender was associated with a higher probability of suffering from anxiety, depression, and stress (30). In addition to policy responses to mitigate the economic and health costs of the pandemic, the disproportionate impact of COVID-19 on the mental health of the most vulnerable members of society requires urgent attention. It is critical that the healthcare system prepare for increased demand for mental healthcare services in both the short and long term, develop innovative solutions to provide care in the context of the pandemic, and prioritize equitable access (40).

In the associative analysis, it was found that there is a significant association between food insecurity and mental health outcomes such as depression, anxiety, and stress. In the adjusted analysis, it was observed that households with mild food insecurity are 2.04 times more likely to have moderate-severe anxiety, highlighting that anxiety is more premature when there is food insecurity. Contrary to these results, Pourmotabbed et al. (21) found no positive association between food insecurity and anxiety. While another study, in adults with very low food security, almost half (49.4%) tested positive for anxiety (41) and even adults with very low food security were 6.19 times more likely to test positive for anxiety (42).

At higher levels of food insecurity (moderate and severe) the odds of having anxiety, depression, and stress increased substantially by up to 10.44, 5.7, and 8.5 times the odds of having anxiety, depression and moderate-severe stress, respectively. These results are consistent with other studies where a clear pattern was evident with increasing levels of food insecurity associated with higher odds of depression, anxiety, and high stress, compared to food secure individuals (43, 44). Because of the strong associations between food security and emotional state disturbances, policies to mitigate, and prevent food insecurity may also have mental health benefits by alleviating stress, depression, and anxiety about practical concerns related to one’s ability to secure sufficient food (45, 46).

Importantly, these findings demonstrate how high rates of food insecurity and mental health persist during the pandemic in the Peruvian population. Even when the economy begins to recover. There is a need to review the temporary policies currently in place imposed by governments in a way that ensures adequate access to food and medical care, in addition to focusing on the creation of affordable healthcare plans that cover mental healthcare (41).

This study has several limitations. First, although the study is designed to be nationally representative, our sample differs in some respects from the general adult population, in particular, there is an overrepresentation of coast region (54.9%), women (68%), and socioeconomic levels C and D, therefore, the results must be taken with caution since they do not represent the Peruvian population. Second, the data was collected through an online survey, thus excluding those without Internet access. The survey was also conducted only in Spanish, so it did not capture non-Spanish speakers, such as the Quechua-speaking populations of the Andes or the indigenous peoples of the jungle, which limits the generalizability of the results to these populations. Finally, the cross-sectional design does not allow us to determine causality.

## Conclusion

In conclusion, this study demonstrates that food insecurity during the COVID-19 pandemic is associated with mental health disturbances in the Peruvian population. Therefore, there is a need for continued evaluation of the immediate impact of COVID-19, its new variants, and the long-term health implications of barriers to food access, diet quality, and population nutrition.

## Data availability statement

The raw data supporting the conclusions of this article will be made available by the authors, without undue reservation.

## Ethics statement

The studies involving human participants were reviewed and approved by the Ethics and Research Committee of the Universidad Peruana Unión (No. 2021-CE-EPG-000014). The patients/participants provided their written informed consent to participate in this study.

## Author contributions

MA-C and EN-N conceived the idea of this project, participated in data collection, and wrote the initial draft of the manuscript. SH-V participated in analysis and reviewed and edited the manuscript. EN-N, WM-G, and CG were collaborators with MA-C at each step and reviewed and edited the manuscript. All authors approved the final version of the manuscript.

## References

[1] Shaw DJ. World food summit, 1996. In: Shaw DJ editor. *World Food Security: A History Since 1945*. London: Palgrave Macmillan UK (2007). p. 347–60.

[2] FAO, FIDA, OMS, PMA Y UNICEF. *El Estado De La Seguridad Alimentaria Y La Nutrición En El Mundo.* Rome: FAO (2021).

[3] Ayele AW Kassa M Fentahun Y Edmealem H. Prevalence and associated factors for rural households food insecurity in selected districts of East Gojjam Zone, Northern Ethiopia: cross-sectional study. *BMC Public Health.* (2020) 20:202. 10.1186/s12889-020-8220-0 32033552 PMC7007667

[4] Gundersen C Hake M Dewey A Engelhard E. Food insecurity during covid-19. *Appl Econ Perspect Policy.* (2021) 43:153–61. 10.1002/aepp.13100 33042509 PMC7537061

[5] Rodriguez C Crowder SL Rodriguez M Redwine L Stern M. Food insecurity and the hispanic population during the covid-19 pandemic. *Ecol Food Nutr.* (2021) 60:548–63. 10.1080/03670244.2021.1974014 34617866 PMC9614706

[6] Pillaca S Villanueva M. Evaluación de la seguridad alimentaria y nutricional en familias del distrito de los morochucos en ayacucho, Perú. *Rev Perú Med Exp Salud Publica.* (2015) 32:73–9. 26102108

[7] World Bank. *Prevalence of Moderate or Severe Food Insecurity in the Population.* (2021). Available online at: https://dataworldbankorg/indicator/SNITKMSFIZS?locations=PE (accessed July 21, 2022).

[8] Santos MP Brewer JD Lopez MA Paz-Soldan VA Chaparro MP. Determinants of food insecurity among households with children in Villa El Salvador, Lima, Peru: the role of gender and employment, a cross-sectional study. *BMC Public Health.* (2022) 22:717. 10.1186/s12889-022-12889-4 35410187 PMC8996213

[9] Pradeilles R Pareja R Creed-Kanashiro HM Griffiths PL Holdsworth M Verdezoto N Diet and food insecurity among mothers, infants, and young children in peru before and during covid-19: a panel survey. *Matern Child Nutr.* (2022) 18:e13343. 10.1111/mcn.13343 35274825 PMC9115223

[10] Castillo AS Flores VAR Chalco FLS Cabrera LCZ. Desarrollo económico y social en el perú en el contexto de la crisis sanitaria del covid-19 y en el marco del bicentenario de la república. *Socialium.* (2022) 6:e1054.

[11] INEI. Instituto Nacional De Estadística E Informática. *Evolución De La Pobreza Monetaria En El Perú 2009-2020.* (2021). Available online at: https://wwwineigobpe/media/MenuRecursivo/boletines/02-informe-tecnico-empleo-nacional-ene-feb-mar-2021pdf (accessed July 21, 2022).

[12] Pereira M Oliveira AM. Poverty and food insecurity may increase as the threat of covid-19 spreads. *Public Health Nutr.* (2020) 23:3236–40. 10.1017/s1368980020003493 32895072 PMC7520649

[13] Olarte-Durand M Roque-Aycachi JB Rojas-Humpire R Canaza-Apaza JF Laureano S Rojas-Humpire A [Mood and sleep quality in peruvian medical students during covid-19 pandemic]. *Rev Colomb Psiquiatr.* (2021). [Epub ahead of print]. 10.1016/j.rcp.2021.11.010 38724170

[14] Chávez Sosa JV Mego Gonzales FM Aliaga Ramirez ZE Cajachagua Castro M Huancahuire-Vega S, editors. Depression associated with caregiver quality of life in post-COVID-19 patients in two regions of Peru. *Healthcare.* (2022) 10:1219. 10.3390/healthcare10071219 35885746 PMC9323236

[15] Piqueras JA Ramos V Martínez AE Oblitas LA. Emociones negativas y su impacto en la salud mental y física. *Suma Psicológ.* (2009) 16:85–112.

[16] Huancahuire-Vega S Newball-Noriega EE Rojas-Humpire R Saintila J Rodriguez-Vásquez M Ruiz-Mamani PG Changes in eating habits and lifestyles in a peruvian population during social isolation for the covid-19 pandemic. *J Nutr Metab.* (2021) 2021:4119620. 10.1155/2021/4119620 34868677 PMC8633849

[17] Villarreal-Zegarra D Copez-Lonzoy A Vilela-Estrada AL Huarcaya-Victoria J. Depression, post-traumatic stress, anxiety, and fear of covid-19 in the general population and healthcare workers: prevalence, relationship, and explicative model in Peru. *BMC Psychiatry.* (2021) 21:455. 10.1186/s12888-021-03456-z 34530803 PMC8445782

[18] Ruiz-Frutos C Palomino-Baldeón JC Ortega-Moreno M Villavicencio-Guardia MDC Dias A Bernardes JM Effects of the covid-19 pandemic on mental health in Peru: psychological distress. *Healthcare.* (2021) 9:691. 10.3390/healthcare9060691 34201042 PMC8227219

[19] Polsky JY Gilmour H. Food insecurity and mental health during the covid-19 pandemic. *Health Rep.* (2020) 31:3–11. 10.25318/82-003-x202001200001-eng 33325672

[20] Elgar FJ Pickett W Pförtner TK Gariépy G Gordon D Georgiades K Relative food insecurity, mental health and wellbeing in 160 countries. *Soc Sci Med.* (2021) 268:113556. 10.1016/j.socscimed.2020.113556 33293171

[21] Pourmotabbed A Moradi S Babaei A Ghavami A Mohammadi H Jalili C Food insecurity and mental health: a systematic review and meta-analysis. *Public Health Nutr.* (2020) 23:1778–90. 10.1017/s136898001900435x 32174292 PMC10200655

[22] Heuveline P Tzen M. Beyond deaths per capita: comparative covid-19 mortality indicators. *BMJ Open.* (2021) 11:e042934. 10.1136/bmjopen-2020-042934 33692179 PMC7948156

[23] Chauvin L. Peruvian covid-19 vaccine scandal spreads. *Lancet.* (2021) 397:783. 10.1016/s0140-6736(21)00508-0 PMC7946602 33640052

[24] Romero OEV Romero FMV. Evaluación del nivel socioeconómico: presentación de una escala adaptada en una población de lambayeque. *Rev Cuerpo Méd Hosp Nac Almanzor Aguinaga Asenjo.* (2013) 6:41–5.

[25] Segall Corrêa AM Álvarez Uribe MC Melgar Quiñonez H Pérez Escamilla R. *Escala Latinoamericana Y Caribeña De Seguridad Alimentaria (Elcsa): Manual De Uso Y Aplicaciones.* Rome: UN Food and Agricultural Organization (2012).

[26] Muñoz-Astudillo MN Martínez JW Quintero AR. [Validating Latin-American and Caribbean Latin-American food security scale on pregnant adolescents]. *Rev Salud Publica.* (2010) 12:173–83. 10.1590/s0124-00642010000200001 21031228

[27] Pérez-Escamilla R Dessalines M Finnigan M Pachón H Hromi-Fiedler A Gupta N. Household food insecurity is associated with childhood malaria in rural Haiti. *J Nutr.* (2009) 139:2132–8. 10.3945/jn.109.108852 19741201

[28] Silva JLC Sánchez JAP Sánchez AP. La escala latinoamericana y del caribe sobre seguridad alimentaria (ELCSA): una herramienta confiable para medir la carencia por acceso a la alimentación/the Latin American and caribbean food security scale (ELCSA): a reliable tool to measure lack access to food. *RICSH Revista Iberoamericana Ciencias Soc Human.* (2017) 6:263–86.

[29] Madrid MS Carrascal JM Castro ÁM. Escalas para estudiar percepción de estrés psicológico en el climaterio. *Revista Ciencias Biomédicas.* (2013) 4:318–26.

[30] Ozamiz-Etxebarria N Dosil-Santamaria M Picaza-Gorrochategui M Idoiaga-Mondragon N. Níveis de estresse, ansiedade e depressão na primeira fase do surto de covid-19 em uma amostra no norte da espanha. *Cad. Saúde Pública.* (2020) 36:e00054020. 10.1590/0102-311X00054020 32374806

[31] Lima MG Valdez IC. Impacto de la pandemia covid-19, en la salud mental de pacientes que acuden a una clínica privada en ventanilla. *Revista Científica Ágora.* (2020) 7:114–9.

[32] Kent K Murray S Penrose B Auckland S Visentin D Godrich S Prevalence and socio-demographic predictors of food insecurity in Australia during the covid-19 pandemic. *Nutrients.* (2020) 12:2682. 10.3390/nu12092682 32887422 PMC7551067

[33] Niles MT Bertmann F Belarmino EH Wentworth T Biehl E Neff R. The early food insecurity impacts of covid-19. *Nutrients.* (2020) 12:2096. 10.3390/nu12072096 32679788 PMC7400862

[34] Robayo CV Iza PI Mejía CM. Inseguridad alimentaria en hogares ecuatorianos durante el confinamiento por covid-19. *Invest Desarroll.* (2020) 12:9–15.

[35] Chen MA Grapsa E Ismail G Rogan M Valdivia M Alfers L Covid-19 Y trabajo informal: evidencia de once ciudades. *Revista Internacional del Trabajo.* (2022) 141:33–65.

[36] Flores Arocutipa JP Fernández Sosa LE. Efectos del coronavirus covid-19 en el empleo y los ingresos familiares en sur del Perú, 2020. *Revista Venezolana de Gerencia.* (2022) 27:299–318.

[37] Elsahoryi N Al-Sayyed H Odeh M McGrattan A Hammad F. Effect of covid-19 on food security: a cross-sectional survey. *Clin Nutr ESPEN.* (2020) 40:171–8. 10.1016/j.clnesp.2020.09.026 33183533 PMC7533117

[38] Food and Agriculture Organization [FAO]. *Seguridad Alimentaria Bajo La Pandemia De Covid-19.* Rome: Food and Agriculture Organization (2020).

[39] Zabetakis I Lordan R Norton C Tsoupras A. Covid-19: the inflammation link and the role of nutrition in potential mitigation. *Nutrients.* (2020) 12:1466. 10.3390/nu12051466 32438620 PMC7284818

[40] Aulestia-Guerrero EM Capa-Mora ED. Una mirada hacia la inseguridad alimentaria sudamericana. *Ciência Saúde Coletiva.* (2020) 25:2507–17. 10.1590/1413-81232020257.27622018 32667535

[41] Sundermeir SM Wolfson JA Bertoldo J Gibson DG Agarwal S Labrique AB. Food insecurity is adversely associated with psychological distress, anxiety and depression during the covid-19 pandemic. *Prev Med Rep.* (2021) 24:101547. 10.1016/j.pmedr.2021.101547 34518794 PMC8425295

[42] Wolfson JA Garcia T Leung CW. Food insecurity is associated with depression, anxiety, and stress: evidence from the early days of the covid-19 pandemic in the United States. *Health Equity.* (2021) 5:64–71. 10.1089/heq.2020.0059 33681691 PMC7929913

[43] Fang D Thomsen MR Nayga RM Jr. The association between food insecurity and mental health during the covid-19 pandemic. *BMC Public Health.* (2021) 21:607. 10.1186/s12889-021-10631-0 33781232 PMC8006138

[44] Zahidi F Khalid M Surkan PJ Azadbakht L. Associations between food insecurity and common mental health problems among reproductive-aged women in Kabul-Afghanistan. *Front Nutr.* (2021) 8:794607. 10.3389/fnut.2021.794607 35047547 PMC8761756

[45] Wolfson JA Leung CW. Food insecurity and covid-19: disparities in early effects for us adults. *Nutrients.* (2020) 12:1648. 10.3390/nu12061648 32498323 PMC7352694

[46] Riehm KE Holingue C Smail EJ Kapteyn A Bennett D Thrul J Trajectories of mental distress among U.S. adults during the covid-19 pandemic. *Ann Behav Med.* (2021) 55:93–102. 10.1093/abm/kaaa126 33555336 PMC7929474

